# A case of sporotrichosis caused by different *Sporothrix brasiliensis* strains: mycological, molecular, and virulence analyses

**DOI:** 10.1590/0074-02760190260

**Published:** 2019-10-21

**Authors:** Manoel Marques E Oliveira, Rodrigo Almeida-Paes, Danielly Corrêa-Moreira, Cintia de Moraes Borba, Rodrigo Caldas Menezes, Dayvison Francis Saraiva Freitas, Antonio Carlos Francesconi do Valle, Armando de Oliveira Schubach, Monica Bastos de Lima Barros, Joshua D Nosanchuk, Maria Clara Gutierrez-Galhardo, Rosely Maria Zancopé-Oliveira

**Affiliations:** 1 Fundação Oswaldo Cruz-Fiocruz, Instituto Nacional de Infectologia Evandro Chagas, Laboratório de Pesquisa Clínica em Dermatozoonoses em Animais Domésticos, Rio de Janeiro, RJ, Brasil; 2 Fundação Oswaldo Cruz-Fiocruz, Instituto Nacional de Infectologia Evandro Chagas, Laboratório de Micologia, Rio de Janeiro, RJ, Brasil; 3 Fundação Oswaldo Cruz-Fiocruz, Instituto Oswaldo Cruz, Laboratório de Taxonomia, Bioquímica e Bioprospecção de Fungos, Rio de Janeiro, RJ, Brasil; 4 Fundação Oswaldo Cruz-Fiocruz, Instituto Nacional de Infectologia Evandro Chagas, Laboratório de Pesquisa Clínica em Dermatologia Infecciosa, Rio de Janeiro, RJ, Brasil; 5 Fundação Oswaldo Cruz-Fiocruz, Instituto Nacional de Infectologia Evandro Chagas, Laboratório de Vigilância em Leishmaniose, Rio de Janeiro, RJ, Brasil; 6 Fundação Oswaldo Cruz-Fiocruz, Escola Nacional de Saúde Pública, Rio de Janeiro, RJ, Brasil; 7 Albert Einstein College of Medicine, New York, United States of America

**Keywords:** Sporothrix, genotypic analyses, PCR fingerprint, experimental murine model, virulence

## Abstract

**BACKGROUND:**

Sporotrichosis is a subcutaneous mycosis caused by dimorphic pathogenic fungi belonging to the *Sporothrix* genus. Pathogenic *Sporothrix* species typically produce melanin, which is known to be a virulence factor.

**OBJECTIVES:**

The aim of this study was to perform phenotypic, genotypic, and virulence analyses of two distinct *Sporothrix brasiliensis* strains isolated from the same lesion on a patient from Rio de Janeiro.

**METHODS AND FINDINGS:**

Genotypic analyses by partial sequencing of the *calmodulin*, *β-tubulin*, and *chitin synthase* genes, as well as polymerase chain reaction (PCR)-fingerprinting by T3B, M13, and GACA, showed that the isolates were very similar but not identical. Both isolates had similar phenotypic characteristics and effectively produced melanin in their yeast forms, accounting for their ability of causing disease in a murine sporotrichosis model. Remarkably, isolate B was albino in its environmental form but caused more severe disease than the pigmented A isolate.

**CONCLUSIONS:**

These findings indicate that the patient was infected by two genetically and biologically distinct *S. brasiliensis* that vary in their production of melanin in their environmental forms. The results underscore the importance of characterizing phenotypically different isolates found in the same clinical specimen or patient.

Sporotrichosis is a globally distributed subcutaneous mycosis that usually involves the skin and subcutaneous tissues but occasionally spreads to internal organs, especially in immunocompromised patients.^1^ In the last decade, new *Sporothrix* species have been described,^2^ ^,^ ^3^ expanding the of the species among the medically relevant agents of sporotrichosis to *S. brasiliensis*, *S. globosa*, *S. luriei*, *S. mexicana*, and *S. schenckii sensu stricto*.^2^ ^,^ ^4^ ^,^ ^5^ Other environmental species, such as *S. pallida* and *S. chilensis* have also recently been identified as the less common causative agents of the disease.^2^ ^,^ ^3^ ^,^ ^6^ ^,^ ^7^

*S. schenckii sensu lato* can synthesize three different types of melanin, both in the yeast and mycelial forms *in vitro*.^8^ Furthermore, yeast cells in the infected tissues are also melanized, suggesting that melanin participates in fungus-host interactions.^9^ In fact, melanin protects diverse fungi from the immune defence of the host.^10^ Fungal melanins are complex polymers with covalently linked aromatic subunits that are produced by different synthetic pathways, known as the 1,8-dihydroxynaphthalene (DHN), dihydroxyphenylalanine, and L-tyrosine pathways depending on the species.^11^ Melanin functions in *Sporothrix* spp. include protection against oxygen and nitrogen free radicals, resistance against UV light and antifungal drugs, such as amphotericin B and terbinafine and evasion from macrophages.^12^ ^,^ ^13^

Since 1998, the metropolitan Rio de Janeiro area in Brazil has been considered an endemic region for zoonotic sporotrichosis.^14^ Infected cats may carry *Sporothrix* yeast cells on their nails and in the oral cavity. Thus, they can pass the infection to humans through scratching or biting.^15^ Generally, the manifestation of the feline disease precedes the human and canine cases, and the individuals most frequently affected are housewives taking care of cats with sporotrichosis. Several clinical forms have been described in this ongoing epidemic, in which the lymphocutaneous and fixed cutaneous form has been observed in the majority of the cases.^1^ ^,^ ^14^ ^,^ ^15^ However, several uncommon clinical manifestations of the disease have also been described, including high severity requiring hospitalization for intravenous antifungal therapy and even causing mortalities among patients with no obvious immunodeficiencies.^16^ Using the new taxonomic identification methods for the *Sporothrix* species, our group has reported the isolation of *S. schenckii*, *S. globosa*, and *S. brasiliensis* from patients in Rio de Janeiro.^4^ The most frequently identified species in this region is *S. brasiliensis*, which is responsible for ≥ 83% of cases.^4^

During the evaluation of a patient with the disseminated cutaneous form of sporotrichosis from Rio de Janeiro, we identified that she was infected with fungi that generated colonies upon cultivation at room temperature (20-25ºC) that displayed distinct phenotypes, pigmented and albino. Information about this patient has already been published in a case series.^13^ ^,^ ^17^ Briefly, the patient was a 63-year-old woman from a hyperendemic area of sporotrichosis in Rio de Janeiro. She was presented to our institute with approximately 40 disseminated cutaneous nodular and ulcerative lesions after being bitten by a cat with multiple mucocutaneous wounds that were presumably secondary to sporotrichosis. Testing at that time led to the identification of *S. schenckii sensu lato* from the tissue samples. Accordingly, cutaneous disseminated sporotrichosis was diagnosed, and treatment was initiated. The patient had a slow initial response to itraconazole at doses up to 400 mg/day over a period of 6 months. Consequently, a combination of fluconazole and itraconazole (200 mg/day each) was administered for three more months. Due to the presence of many bulky lesions, we also chose to perform monthly curettage of the soft, larger nodular ulcerated lesions to accelerate their disinfection. The patient’s lesions resolved, and she appeared cured after nine months of treatment, and thus the antifungal therapy was stopped. However, in less than two months, a single nodular ulcerated lesion (2 × 1 cm) on the right forearm appeared. She rejected having had any new contact with an animal. This lesion was curetted for culture, and no systemic treatment was administered as the wound resolved over three weeks, prior to her next appointment. The patient was followed up for an additional year, and there was no additional recurrence. However, the culture from the recurrent lesion yielded two dissimilar yeast colonies; one of them was melanized, whereas the other one was not pigmented (albino). In this study, we performed the genotypic, phenotypic, and virulence analyses of these isolates to better understand the differences between them and their relevance to the pathogenesis of the disease.

## MATERIALS AND METHODS

*Ethics statement* - This study was approved by the Research Ethics Committee of INI/Fiocruz (CAAE 28063114.2.0000.5262) and by the IOC Ethics Commission for the Use of Laboratory Animals-Fiocruz (CEUA L-022/2015).

*Isolates* - Two macroscopically distinct isolates were recovered from a single lesion on the patient. At room temperature, they were noted to be a melanin-producing isolate (IPEC18782A, hereafter named isolate A) and an albino (amelanotic) isolate (IPEC18782B, hereafter named isolate B). These isolates were initially identified as *S. schenckii sensu lato* by conventional methods as described by Dixon et al.^18^ Five strains were used as controls for phenotypic and genotypic characterization, and these included the type strain of *S. brasiliensi*s CBS120339 (formerly IPEC 16490)^2^ and the reference strains IPEC 27722 (*S. schenckii)*, IPEC 27135 (*S. globosa*), SPA8 (*S. pallida*), and MUM 11.02 (*S. mexicana*).^4^ ^,^ ^5^

*Species identification* - A detailed phenotypic analysis was performed to determine the species of the clinical isolates and control strains. Molecular methods were applied to confirm the results of the phenotypic tests^5^ ^,^ ^7^ as described below.

*Molecular procedures* - Genomic DNA was extracted from the *Sporothrix* mycelial form previously.^4^ Polymerase chain reaction (PCR) fingerprinting was performed using *M13* (5ʹ-GAGGGTGGCGGTTCT-3ʹ), (*GACA*)_*4*_ , and *T3B*(5-AGGTCGCGGGTTCGAATCC-3ʹ) primers to confirm phenotypic identification and verify similarities between the melanized and albino *Sporothrix* isolates.^7^ ^,^ ^19^ Partial sequencing of the *b-tubulin*,^2^ *calmodulin* (*CAL*) encoding gene,^4^ and *chitin synthase* (*CHS*)^2^ encoding genes and internal transcribed spacer (ITS)^20^ was performed to confirm the cryptic species identification made via the phenotypic tests^2^ ^,^ ^4^ and to further differentiate the two isolates (A and B).

*Antifungal susceptibility* - The susceptibility of the *Sporothrix* isolates to amphotericin B, ketoconazole, itraconazole, voriconazole, posaconazole, and terbinafine was tested using the microdilution method for antifungal susceptibility of filamentous fungi as described by the Clinical and Laboratory Standards Institute in the M38-A2 document.^21^ Each antifungal agent was serially diluted from 0.015 to 8 μg/mL in a sterile 96-well round bottom plate. *Aspergillus flavus* (ATCC 204304) and *Aspergillus fumigatus* (ATCC 204305) were used as controls in each plate. A final inoculum of 2 × 10^4^ to 1 × 10^5^ conidia/mL was used to inoculate the antifungal plates. The plates were incubated at 37ºC for 72 h. The readings were visually made by verifying any turbidity in the inoculated wells and comparing them with the positive control, which was free of antifungal drugs, and the negative control, which consisted of RPMI 1640 medium alone. For amphotericin B, itraconazole, posaconazole, and voriconazole, the minimum inhibitory concentration (MIC) was the lowest concentration that produced complete inhibition of growth. For ketoconazole, the MIC was the lowest concentration producing a 50% reduction in growth, and it was the lowest concentration for terbinafine, producing at least an 80% reduction in growth.

*Quantification of the putative virulence factors* - Expression of several virulence-related phenotypes by the clinical isolates was assessed. Protease production was evaluated on minimal medium (15 mM glucose, 10 mM MgSO_4_, 29.4 mM K_2_HPO_4_,13 mM glycine, and 3.0 mM thiamine; pH 4.5) supplemented with 0.1% azoalbumin. Urease production was evaluated on Christensen urea broth (1 g/L peptone, 5 g/L NaCl, 2 g/L KH_2_PO_4_, 20 g/L urea, 1 g/L dextrose, and 0.0016% phenol red). Lipase production was studied on rhodamine B agar plates (7 g/L YNB, 20 mL/L olive oil, 50 mL/L fetalfetal bovine serum, 1 mM rhodamine B). Melanin production was studied as previously described by our group,^8^ using a minimal medium (15 mM glucose, 10 mM MgSO_4_, 29.4 mM KH_2_PO_4_, 13 mM glycine, and 3.0 mM thiamine; pH 5.5) supplemented with or not without 1 mM L-DOPA or 10 mM L-tyrosine. DHN- and L-DOPA melanins were studied after the denaturant, hot-acid treatment of the cultures to yield melanin ghosts from the isolates.^8^ Pyomelanin levels was studied ion the supernatants of the cultures grown in the presence of L-tyrosine. A semi-quantitative analysis based on the absorbance of the supernatants at 340 nm.^22^

Experimental inoculation in mice

*Mice* - Ninety male BALB/c mice weighing approximately 25 g were used in accordance with the requirements of the IOC Ethics Commission for the Use of Laboratory Animals (license L-022/2015). Of these mice, eight were used for fungal reactivation and 82 for virulence assays. Eighty-two mice were divided into groups and inoculated with the two *Sporothrix* isolates as follows: for each isolate (A and B), four mice were inoculated for fungal reactivation. A total of 30 mice, subdivided into three groups of 10 animals, were inoculated with the isolates individually (A or B) or in combination (A and B) to evaluate the clinical signs of the disease and the survival curve. The combination of the isolates was aimed at reproducing the clinical situation of the patient since she had a lesion with the two isolates. An additional 30 mice were divided into groups and inoculated in the same manner as described above and used for tissue burden and histopathological studies. A control group (14 mice) was inoculated with phosphate-buffered saline (PBS). The mice received food and filtered water *ad libitum*.

*Fungal reactivation* - *Sporothrix* isolates A and B were cultured in Sabouraud broth (Difco^TM^ Becton, Dickinson and Company/Sparks, MD 21152 USA) at 25ºC with shaking at 100 oscillations/min. After 11 days, the conidia were collected, counted with a Neubauer chamber, and their viability was determined by the colony-forming unit (CFU) protocol.^23^ The fungal reactivation group of eight mice was subdivided into two groups of four animals and intraperitoneally inoculated with 3 × 10^6^ conidia of each isolate in 0.02 mL of sterile PBS. After 20 days, the mice were subjected to euthanasia by prolonged CO_2_ exposure, and the isolates were recovered through the culture of the spleens on Mycosel agar (Becton, Dickinson and Company, Sparks, MD, USA) at 37ºC.

*Fungal inoculation* - Conidia were obtained as described above for fungal reactivation. Three groups of 10 mice were subcutaneously inoculated in the tail basis region with 3.2 × 10^5^ conidia (> 87% viable) of isolate A and B alone or together (1.6 × 10^5^ conidia of each) in 0.02 mL of sterile PBS. A control group was similarly injected with PBS.

*Euthanasia, necropsy, CFU determination, and splenic index* - Three mice from each group were weighed, euthanized by prolonged CO_2_ exposure, and necropsied 21, 35, and 49 days after fungal inoculation. After a macroscopic examination of the organs, the spleen, lungs, kidneys, heart, and liver were aseptically removed. The spleens were weighed and homogenized in sterile PBS to determine the number of viable fungal cells. The suspension was adjusted to 30 mg of tissue/mL, and 100 µL of each homogenate was spread on a Petri dish containing Mycosel agar (Becton, Dickinson and Company, Sparks, MD, USA), incubated at 37ºC for 15 days, and then the fungal colony number was determined.^24^ The spleen and body weight ratios of each infected and control mice were also determined. The ratios of the relative weight of spleens from infected mice were expressed as units in relation to the control. The mean value for the relative weight of spleens in each control group of mice was considered to be equal to one unit.^25^

*Histopathology* - After macroscopic examination of the organs during necropsy, the livers, lungs, kidneys, and hearts were immediately fixed in 10% buffered formalin, embedded in paraffin, sectioned and placed on slides, and stained with haematoxylin-eosin (HE) or Grocott methenamine silver (GMS). The inflammatory infiltrate was classified as granulomatous, pyogranulomatous, or non-granulomatous if macrophages, macrophages alongside numerous neutrophils, or other cell types predominated, respectively.^26^ The non-granulomatous infiltrate was classified as suppurative if it contained neutrophils and non-suppurative if it was primarily composed of mononuclear cells, such as lymphocytes, plasma cells, and macrophages. The granulomatous or pyogranulomatous inflammation was classified as well-organized (nodular) or poorly-organized (diffuse).^26^ The distribution of the inflammatory infiltrate was classified as: focal (one inflammatory focus), multifocal (more than one inflammatory focus), and diffuse (inflammatory cells evenly distributed in the tissue section). For the evaluation of inflammatory intensity, the sum of all cell types (macrophages, plasma cells, lymphocytes, eosinophils, and neutrophils) and the number of well-organised granulomas detected in the inflammatory infiltrate were calculated. For this purpose, a 1-mm^2^ optical grid and manual cell counter were used. The total number of inflammatory cells was determined in HE-stained sections in one microscopic field at × 400 magnification, and the number of well-organized granulomas in five microscopic fields at × 100 magnification. Both assessments were performed in the most cellular area of the histological sections. Intra-lesional yeast-like forms were quantitated in GMS-stained sections using the same method described above for the inflammatory cells. According to the sum of all cell types detected in an inflammatory infiltrate and the number of granulomas, the inflammatory intensity was classified as absent, mild (1-110 inflammatory cells and < 10 well-organized granulomas/mm^2^), moderate (111-350 inflammatory cells and < 10 well-organized granulomas/mm^2^), and severe (> 350 inflammatory cells or ≥ 10 well-organized granulomas/mm^2^). The distribution of pericardial mineralization and fibrosis was classified as focal (one focus of mineralization and fibrosis) or multifocal (> one focus of mineralization and fibrosis). The spleen was removed for the determination of splenic index and CFUs. The same procedure was followed for the control group.

*Data analysis* - Sequences from both DNA strands were generated and edited with the Sequencher 4.6 software package (Genes Codes Corporation, USA), followed by alignment by means of the Mega version 4.0.2 software. Species identification was performed by searching databases with the BLAST (Basic Local Alignment Search Tool- NIH). A Bootstrap test with 1,000 replicates was conducted for both Neighbour-joining analyses.^4^ PCR fingerprinting profiles were analysed by using the software Bionumerics (version 5.1; Applied Maths BVBA, Sint-Martens-Latem, Belgium). Comparisons between groups for the CFU, weight loss, and splenic index experiments were analysed by the ANOVA test. Survival data were analysed using Kaplan Meier survival plots followed by log-rank tests. Data with a p ≤ 0.05 were considered significant.

## RESULTS

**Fig. 1:**
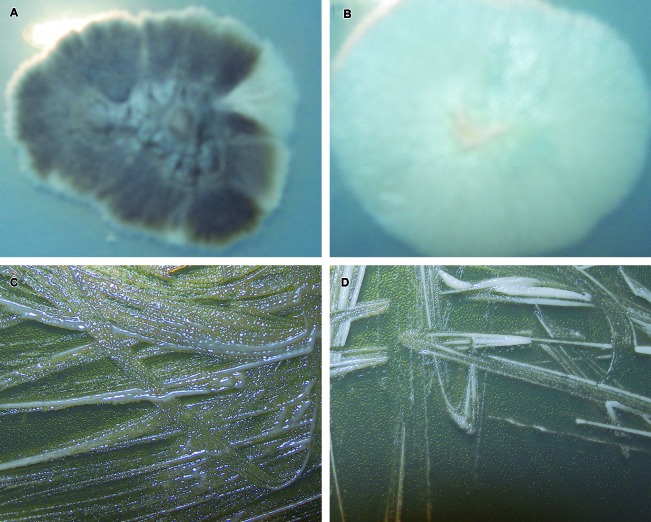
macroscopic features of the melanotic and amelanotic *Sporothrix* strains isolated from a patient with sporotrichosis: (A) growth of the melanotic strain and (B) the amelanotic strain on potato dextrose agar after 15 days of incubation at 30ºC; (C) growth of the melanotic strain and (D) amelanotic strain on brain heart infusion agar after seven days of incubation at 37ºC.

*Phenotypic tests* - Table I shows the mycological aspects (phenotypic and genotypic) of the two *S. schenckii sensu lato* isolates derived from the clinical specimen of a single lesion on the patient’s limb. Additionally, Fig. 1A-B shows the macroscopic characteristics of these isolates at 30ºC, showing a melanin-producing profile in isolate A and an amelanotic profile in isolate B. At 30ºC, both yielded colonies that microscopically consisted of hyaline, septate hyphae with conidiophores rising at right angles with sympodial conidia. The melanized isolate presented with abundant dematiaceous conidia formed along the hyphae. These structures were not observed on the amelanotic isolate. The isolates could both grow at 37ºC (Fig. 1C-D), yielding budding-yeast cells on BHI Agar. The melanized isolate (A) presented phenotypical characteristics consistent with *S. brasiliensis* (Table I). On the other hand, the amelanotic isolate (B) also had all the nutritional requirements and growth rate characteristics of *S. brasiliensis*, except for the absence of dematiaceous conidia, which led to its identification as *Sporothrix* spp.

**TABLE I:** Phenotypic and genotypic characteristics of *Sporothrix* species complex isolated from a single lesion. Final identification to species level by phenotypic and genotypic tests

			Strains		
			IPEC18782A (strain A)	IPEC18782B (strain B)	*S. brasiliensis* (type)
Phenotyping conidia	Hyaline	Hyaline	absent	present	absent
	Dematiaceous	Elongated	absent	absent	absent
		Triangular	absent	absent	present
		Globose	present	absent	absent
Phenotyping assimilation test	Glucose		+	+	+
	Sucrose		-	-	-
	Raffinose		-	-	-
	Range	30ºC	35	22	35
		37ºC	08	02	13
Genotyping	Final identification		*S. brasiliensis*	*S. brasiliensis*	*S. brasiliensis*
	GenBank nº	CAL	HQ426933	HQ426934	AM116899
		Beta	This study	This study	AM116946
		CHS	This study	This study	AM117417

**Fig. 2:**
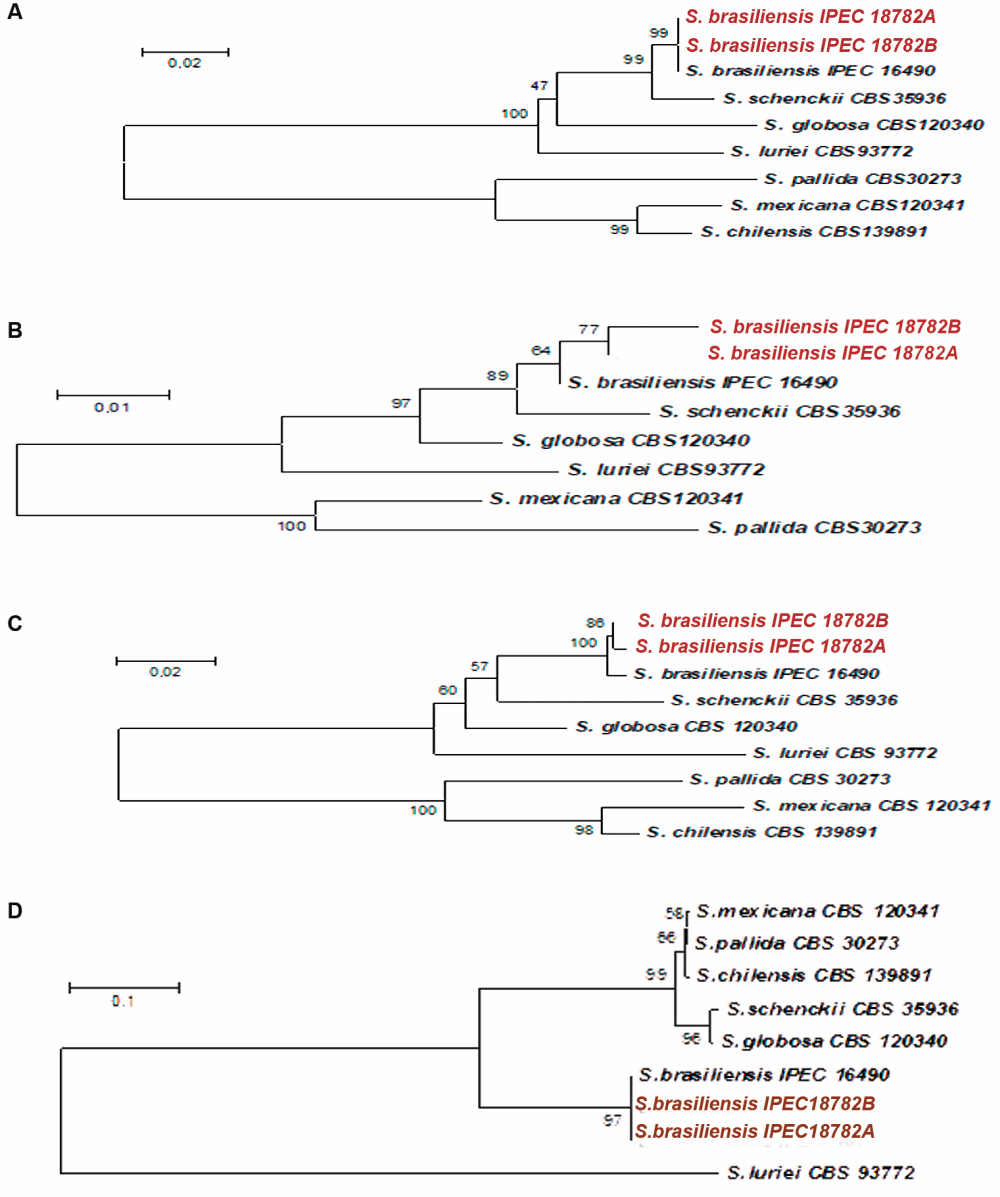
Neighbor-joining phylogram of the partial *CAL* (A), partial *CHS* (B), and partial *β-tubulin* (C) genes obtained from isolates A (18782B) and B (18782A), and the *Sporothrix mexicana*, *S. pallida*, *S. brasiliensis*, *S. schenckii*, and *S. globosa* reference strains constructed with MEGA version 4.0.2. Bootstrap values after 1,000 replicates are presented in the branch node. (D) Comparison of the partial ITS1 and 2 regions of all strains of *S. brasiliensis* demonstrated a high similarity between the type strain IPEC16490 and the patient isolates.

**Fig. 3:**
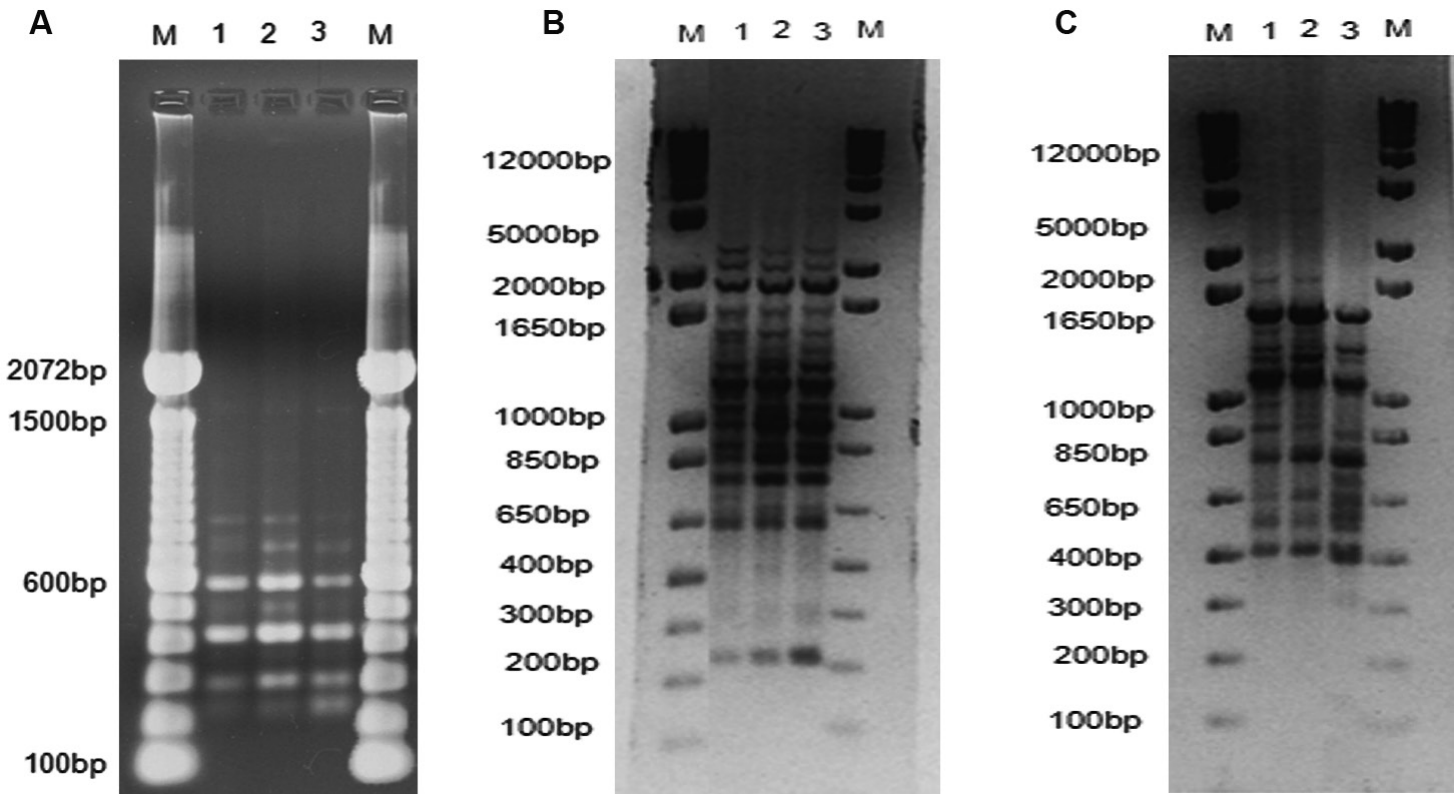
(A) Comparison of the T3B PCR fingerprinting profiles obtained for isolates A and B with the *Sporothrix* reference strains for the main species with clinical association. (1) Isolate A, (2) Isolate B, (3) *S. brasiliensis* (IPEC 16490), (4) *S. globosa* (IPEC 27135), (5) *S. schenckii* (IPEC27722), and (6) negative control. (M) Molecular marker DNA ladder, 100 bp (Invitrogen). (B) Primer-M13-driven DNA fingerprinting profiles of the two isolates included in this study (1) Isolate A and (2) Isolate B, and the reference strain (3) *S. brasiliensis* (IPEC 16490). The DNA molecular marker 1Kb (DNA ladder, Invitrogen) was loaded in the first and last wells. (C) (GACA)_4_-driven DNA fingerprinting profiles of the two isolates (1) Isolate A and (2) Isolate B, and the reference strain (3) *S. brasiliensis* (IPEC 16490). The DNA molecular marker 1Kb (DNA ladder, Invitrogen) was loaded in the first and last wells.

*Molecular studies* - A molecular assessment through PCR fingerprinting and sequencing of partial *CAL* gene was used to confirm the identity of the isolates. The resultant sequences for each isolate were deposited in the GenBank database under accession numbers detailed in Table I. Blast analysis comparing *CAL* sequences was performed, and clustering with the reference strains *S. brasiliensis* CBS120339 (formerly IPEC 16490),^2^ IPEC 27722 (*S. schenckii)*, IPEC 27135 (*S. globosa*), SPA8 (*S. pallida*), and MUM 11.02 (*S. mexicana*) classified both isolates as *S. brasiliensis* (Fig. 2A), and alignment by the Mega Software showed a significant similarity in the partial *CAL* gene between A and B. Furthermore, *b-tubulin*, *CHS*, and *ITS* regions were also sequenced for these isolates and the control strains, and the analyses of the partial sequencing of these four genes demonstrated that the A and B were genetically different, despite being isolated from the same lesion. In the partial *CHS* gene analysis, isolate A showed more similarity with the type *S. brasiliensis* strain and a dissimilar region of diversity when compared with isolate B (Fig. 2B). Analysis of the partial *b-tubulin* gene revealed that isolate B was more similar in the sequence region to the reference strain compared to isolate A (Fig. 2C). Additionally, comparison of the partial ITS1 and two regions all strains of *S. brasiliensis* demonstrated high similarity between the type strain IPEC16490 and the patient isolates (Fig. 2D). Finally, PCR fingerprintings [(M13, (GACA)_4_, and T3B]) confirmed that isolates A and B were very similar, but not identical (Fig. 3A-C).

*Antifungal susceptibility* - The MIC values of both isolates were found to be similar. Both isolates had an MIC of 1.0 µg/mL for both itraconazole and posaconazole (Table II).

**TABLE II:** Minimal inhibitory concentrations (µg/mL) obtained by the broth microdilution method of the two isolates of this study

Strain	18782A	18782B
Amphotericin B	0.5	1.0
Itraconazole	1.0	1.0
Ketoconazole	1.0	0.5
Voriconazole	4.0	2.0
Posaconazole	1.0	1.0
Terbinafine	0.06	0.03

**Fig. 4:**
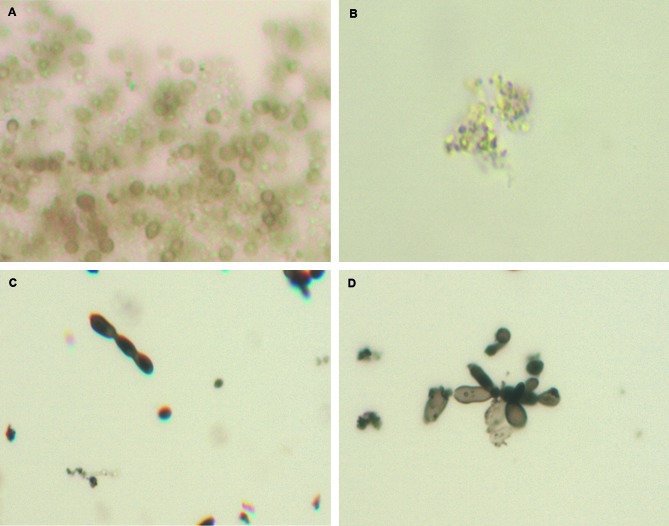
melanin ghosts of the two *Sporothrix* strains isolated from the patient and cultivated on minimal medium without L-DOPA under different conditions. Ghosts derived from (A) the melanotic strain and (B) the amelanotic strain incubated at 30ºC as well as (C) the melanotic strain and (D) the amelanotic strain incubated at 37ºC.

*Quantitation of the virulence factors* - The two *Sporothrix* isolates produced protease, lipase, and urease, with no obvious quantitative difference (data not shown). Regarding melanin production, both isolates could synthesize pyomelanin in both mycelial and yeast forms although pigment production by the mycelial form of the albino B isolate was lower (absorbance at 340 nm = 1.21) than the filamentous dematiaceous A isolate (absorbance at 340 nm = 2.83). Melanin ghosts obtained from isolate A cultivated on minimal medium without L-DOPA had the same shape and size of the fungal conidia, with hyphae completely dissolved by the treatment (Fig. 4A). However, conidia from isolate B were dissolved after denaturant, hot-acid treatment, and only small dysmorphic particles were observed (Fig. 4B). Isolate B hyphae were also completely dissolved by the treatment. Melanin ghosts of these two isolates derived from their yeast forms grown on minimal medium without L-DOPA had similar shapes (Fig. 4C-D). When L-DOPA was added to the cultures, ghosts were similar in shape for both isolates; particles corresponding to the (conidia + hyphae) and cigar-shaped budding yeasts were generated at 30ºC and 37ºC, respectively.

**Fig. 5:**
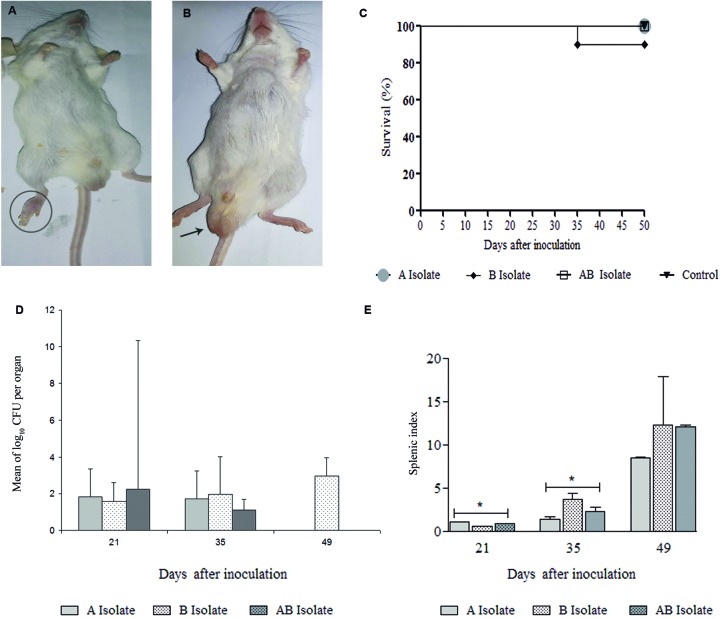
experimental infection. (A-B) Mice inoculated with the *Sporothrix brasiliensis* isolate B, presenting with nodules in the legs (circle) and enlarged testicles (arrow) on the 40th day of infection. (C) The survival of the mice following subcutaneous inoculation of 3.2 × 10^5^ conidia of *S. brasiliensis*. The survival rate of the mice inoculated with isolate B was 90% by day 35 after the inoculation, whereas there was 100% survival in the other groups A, (A + B), and control. The data represent the survival rates of 10 animals per group. The control group was inoculated with phosphate-buffered saline (PBS). p < 0.05. (D) The number of *S. brasiliensis* colony forming units (CFU) isolated from the spleen. Groups of mice were subcutaneously inoculated with 3.2 × 10^5^ conidia. Each point represents the mean number of CFU recovered from the spleens of three mice euthanized at the predetermined days 21, 35, and 49 after the inoculation with isolates A, B, or (A + B). p < .05. (E) The splenic index of the mice following the subcutaneous inoculation of 3.2 × 10^5^ conidia of *S. brasiliensis*, and the control group inoculated with PBS. The spleen and body weight ratio of each infected and control mouse was determined. The ratios of the relative spleen weights of the infected mice were expressed as units in relation to the control. p < 0.5.

*Virulence assessment of the S. brasiliensis isolates* - Mice inoculated with the *S. brasiliensis* isolates (A, B, or A + B) behaved normally and showed no signs of inactivity or weight loss (Table III) during the observed period (49 days). On day 21 post-inoculation, a nodule was detected at the site of inoculation in all the infected mice. By the 40th day of infection, all the mice inoculated with the *S. brasiliensis* isolate B had developed enlarged testicles, and nodules in the legs, tail, and nose (Fig. 5A-B). No additional cutaneous nodules were detected on the mice inoculated with *S. brasiliensis* isolates A or (A + B) by day 49. On necropsy, one mouse belonging to group B was found to have white spots on the heart and many nodules in the liver and spleen (which also was culture-positive). Additionally, this animal had a lesion in the urethra region and its bladder was full of urine with sandy material. None of the mice in groups A or (A + B) had visible nodules in the internal organs.

**TABLE III:** Weight variation (g) of groups of mice inoculated with *Sporothrix brasiliensis* and with phosphate-buffered saline (PBS)

Isolates	Days after inoculation		
	21	35	49
A	↑ 4.29 ± 0.51	↑ 7.19 ± 3.56	↑ 5.61 ± 2.95
B	↑ 4.26 ± 4.82	↑ 2.67 ± 2.24	↑ 6.20 ± 2.26
A+B	↑ 3.29 ± 3.01	↑ 4.72 ± 1.80	↑ 7.87 ± 0.53
Control	↑ 2.97 ± 0.16	↑ 3.49 ± 0.49	↑ 9.07 ± 0.06

Average mouse weight at day 0: 25g; ↑: increase of weight compared to day 0.

*Survival rate of mice inoculated with S. brasiliensis* - The survival rates of mice inoculated with the *S. brasiliensis* isolates are shown in Fig. 5C. There were no deaths in mice inoculated with A or (A + B) isolates, or in the PBS-injected control group during the observation period of 49 days. In contrast, the mice inoculated with isolate B had 10% mortality by day 35 post-infection.

*Fungal burden in the spleens* - The quantitation of the fungal cells from spleens was possible 21 and 35 days after the inoculation for all the isolates. On day 49, fungal cells were recovered only from isolate B were recovered. No statistically significant difference in the recovery of the fungal cells was observed between days 21 and 35 among the three infected groups (Fig. 5D).

*Splenic index* - The mean splenic index values revealed the presence of splenomegaly in all the infected groups (Fig. 5E). The splenomegaly was continuous and the organ reached the largest size 49 days after the inoculation. Statistically significant differences were observed 35 days post-infection in the group inoculated with isolate B. The relative weight of the control spleens was considered one unit.

**Fig. 6:**
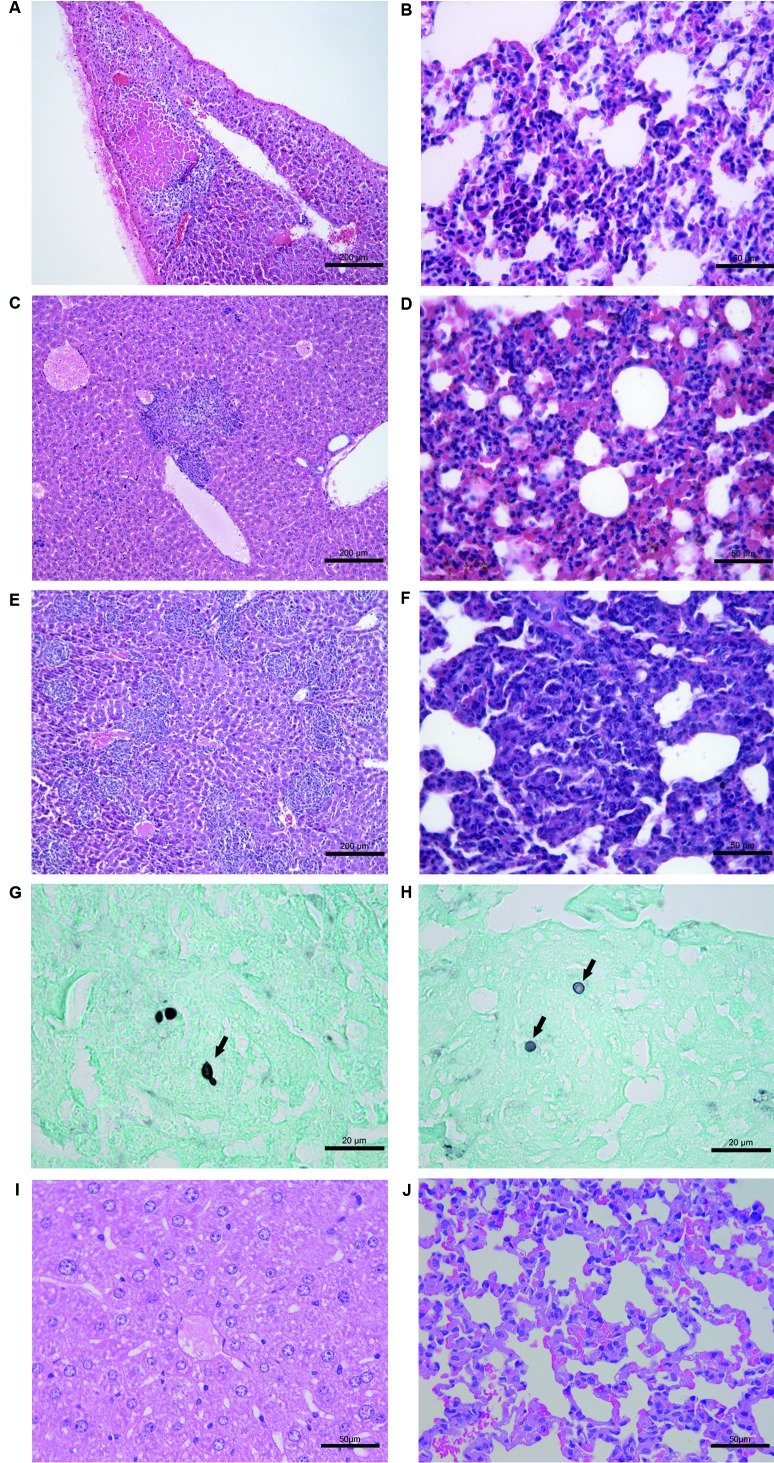
(A-J) Histological findings in the liver and lungs of mice inoculated with the *Sporothrix brasiliensis* isolates. HE. (A) Isolate A, liver, 21 days after the inoculation. Hepatitis, pyogranulomatous, necrotizing, focal, and moderate. (B) Isolate A, lung, 49 days after the inoculation. Pneumonia, pyogranulomatous (poorly organized), diffuse, and moderate. (C) Isolate (A + B), liver, 35 days after the inoculation. Hepatitis, pyogranulomatous, focal, and moderate. A perivascular well-organized granuloma was observed. (D) Isolate (A + B), lung, 49 days after the inoculation. Pneumonia, pyogranulomatous (poorly organized), diffuse, and moderate. (E) Isolate B, liver, 49 days after the inoculation. Hepatitis, pyogranulomatous, multifocal, and severe. Multiple well-organized granulomas were observed. (F) Isolate B, lung, 49 days after the inoculation. Pneumonia, pyogranulomatous, diffuse, and severe. A well-organized granuloma was observed. (G) Isolate B, liver, 49 days after the inoculation. Rare round-to-oval yeasts located in the centre of the granulomas. One of the yeasts shows a narrow-based budding (arrow). GMS. (H) Isolate B, lung, 49 days after the inoculation. Rare round-to-oval yeasts without budding (arrows) were observed in the centre of the granulomas. GMS. (I) Control mice, liver, 49 days after the inoculation, HE; (J) Control mice, lungs, 49 days after the inoculation, HE.

**Fig. 7:**
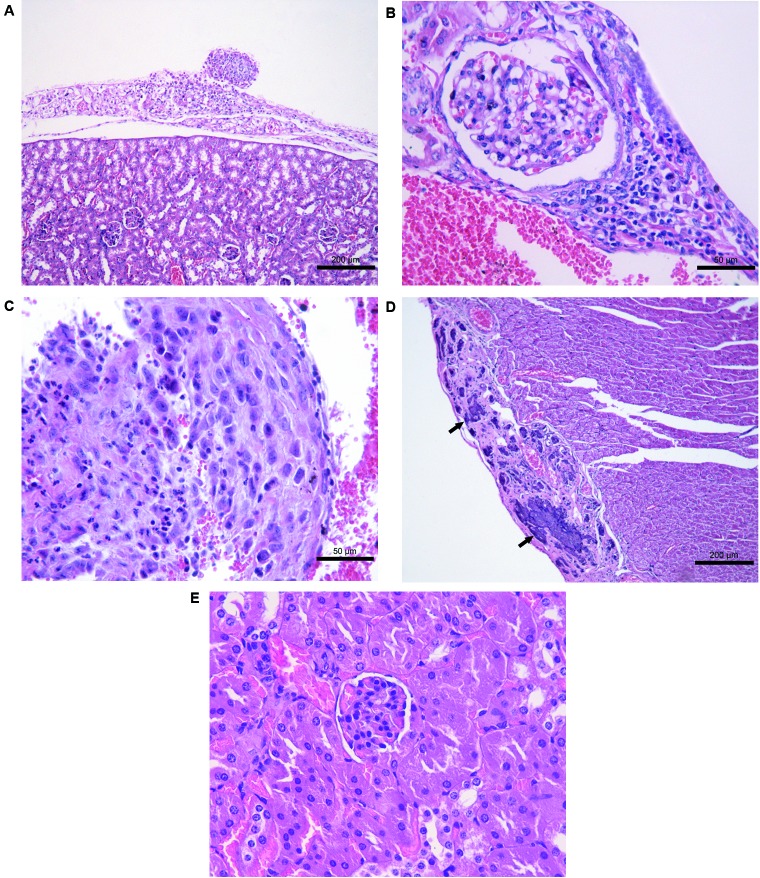
(A-E) Histological findings in the kidneys and heart of the mice inoculated with *Sporothrix brasiliensis* isolates. (A) Isolate B, kidney, 35 days after the inoculation. Pyogranulomatous inflammatory infiltrate in the perirenal adipose tissue. A well-organized granuloma was observed. HE. (B) Isolate (A + B), kidney, 35 days after inoculation. Interstitial nephritis, pyogranulomatous, focal, and mild. HE. (C) Isolate B, heart, 35 days after the inoculation. Suppurative valvular endocarditis, focal, and moderate. HE. (D) Isolate B, heart, 49 days after the inoculation. Pericardial mineralization (arrows) and fibrosis. HE. (E) Control mice, kidney, 35 days after the inoculation, HE.

*Histopathology of the inoculated mice* - The histological alterations in the liver, lungs, kidneys, and hearts of the mice inoculated with the isolates are described in Table IV. Microscopically, a pyogranulomatous or granulomatous inflammatory infiltrate was observed in the following organs: the liver and lungs of the mice inoculated with *S. brasiliensis* isolates A (Fig. 6A-B), A + B (Fig. 6C-D), and B (Fig. 6E-F); the kidneys in the mice inoculated with the *S. brasiliensis* isolates B (Fig. 7A), and A + B (Fig. 7B). In the heart, suppurative valvular endocarditis (Fig. 7C) and pericarditis were observed in the mice inoculated with the isolate B (Table IV). Other alterations included liver necrosis (Fig. 6A) and pericardial mineralization and fibrosis (Fig. 7), which were observed only in the mice inoculated with isolates A and B (Table IV). The combination of isolates A and B in the experimental sporotrichosis model produced disease symptoms similar to those generated by isolate A alone. However, mice inoculated with isolate A were the first to show histological alterations. Additionally, the appearance of pulmonary alterations was accelerated in the mice inoculated with the combination of isolates A and B. These mice were the first to develop pneumonia before it happened in the mice inoculated with isolates A or B alone. Furthermore, unlike isolate A and similar to isolate B, the mice inoculated with both isolates showed renal histological alterations. Spontaneous pericardial mineralization was more severe in the mice inoculated with isolate B than in the mice inoculated with isolate A on day 35 after the inoculation. *Sporothrix* yeasts were observed only in the livers of two mice (Fig. 6) and in the lungs (Fig. 6H) of one mouse inoculated with isolate B. Among these three mice, one showed 1 yeast/mm^2^ in the liver on day 35 after the inoculation, while one showed three yeasts/mm^2^ in the liver, and one showed four yeasts/mm^2^ in the lungs on day 49 after the inoculation. The yeasts were round-to-oval, located in the centre of the granulomas (Fig. 6G-H) and showed a narrow-based budding (Fig. 6G). As expected, the liver, lungs, and kidney of the control mice were histologically normal (Figs 6I-J, 7E).

**TABLE IV:** Histological alterations in the liver, lungs, kidneys and hearts of mice inoculated with the *Sporothrix brasiliensis* isolates A, B and A+B according to the days after inoculation

Organ	Isolate	Days after inoculation		
		21	35	49
Liver	A	Hepatitis, pyogranulomatous, focal or multifocal and moderate. Well organized granulomas.	Hepatitis, pyogranulomatous, necrotizing, focal or multifocal, mild to moderate. Well organized granulomas.	Absent
	B	Absent	Hepatitis, pyogranulomatous, necrotizing, multifocal and moderate. Well organized granulomas.	Hepatitis, pyogranulomatous, multifocal and severe. Well organized granulomas.
	A+B	Absent	Hepatitis, pyogranulomatous, focal and moderate. Well organized granulomas.	Absent
Lungs	A	NP	Absent	Pneumonia, pygranulomatous, diffuse and moderate. Poorly organized granuloma.
	B	wNP	Absent	Pneumonia, pyogranulomatous, diffuse and severe. Well organized granulomas.
	A+B	NP	Pneumonia, pyogranulomatous, multifocal or diffuse and moderate. Poorly organized granuloma.	Pneumonia, pyogranulomatous, diffuse and moderate. Poorly organized granuloma.
Kidney	A	Absent	Absent	Absent
	B	Absent	Pyogranulomatous inflammatory infiltrate, multifocal and moderate in the perirenal adipose tissue. Well organized granuloma.	Pyogranulomatous inflammatory infiltrate, focal and mild in the perirenal adipose tissue. Poorly organized granuloma.
	A+B	Absent	Pyogranulomatous inflammatory infiltrate, focal and mild in the interstitial tissue of cortex and in the perirenal adipose tissue. Poorly organized granuloma.	Absent
Heart	A	NP	Focal pericardial mineralization and fibrosis	Multifocal pericardial mineralization and fibrosis
	B	NP	Suppurative valvular endocarditis and pericarditis, focal and moderate. Multifocal pericardial mineralization and fibrosis	Multifocal pericardial mineralization and fibrosis
	A+B	NP	Absent	Absent

NP: not performed.

## DISCUSSION

Mixed infections with different strains of *Sporothrix* probably occur more commonly than it has been realized, and this phenomenon could cause more severe sporotrichosis as presented here or treatment failure. It is noteworthy that since the onset of the Rio de Janeiro epidemic in 1997-98, more than 5,000 *Sporothrix* spp. isolates have been identified in our laboratory at Fiocruz. This study presents another peculiar aspect of this zoonotic endemic area of sporotrichosis; we presented the first isolation of two different *S. brasiliensis* strains from a single lesion of a patient. In fact, this case is only the second report of a dual-infection by *Sporothrix* spp. A case of sporotrichosis in Japan caused by two different *S. schenckii* strains was described by Kobayashi et al.;^27^ however, the isolates were obtained from separate anatomic sites. Another important difference is that the species involved in our case was *S. brasiliensis*. Since the study of Kobayashi et al.^27^ was performed before the description of the *Sporothrix* genus, it is not possible to determine if their species classification was correct. However, it is not likely that *S. brasiliensis* was the species isolated by them because all the reported cases of sporotrichosis caused by this species are from Brazil.

*S. schenckii sensu lato* can produce melanin in conidia and yeast cells.^8^ ^,^ ^9^ ^,^ ^12^. One of the samples, isolate B, obtained in this study was unable to produce a visible amount of melanin when cultured at 25-30ºC without any phenolic precursor in the growth medium. We found that this isolate is not a truly melanin-deficient mutant, because the treatment of conidia with protease, denaturant, and hot-acid treatments yielded small amounts of dark dysmorphic particles, which were much smaller than those of the type strain *S. brasiliensis* conidia. These particles were similar to the particles we have previously described for a DHN-deficient mutant strain of *S. schenckii*,^22^ and resemble small vesicles or melanosome-like *Candida albicans* ghosts.^9^ In fact, we have recently demonstrated the presence of melanosomes in *S. schenckii*.^8^ We also found that melanin production in the yeast cells of isolate B was higher than that occurred during mycelial growth, and it was comparable to that of isolate A, producing visible melanized colonies. Production of other virulence factors (protease, lipase, and urease) were also comparable among the isolates recovered from the same lesion. Together, these results indicate the virulence potential of both isolates in the parasitic fungal form.

PCR fingerprintings (T3B, M13, and GACA) showed that isolates A and B are very similar, but not identical. Our molecular results together with the other analyses do not permit a conclusion about the origin of the albino isolate. One of the hypotheses is that the patient suffered an injury with a material contaminated with the albino isolate during the course of the treatment of the pre-existing melanized isolate. Alternatively, the initial acquisition could have been with a mixed infection of the two isolates. Another explanation is that a single original melanized strain underwent microevolutions during a long period of parasitism, to the human host and, thereafter losing the ability to produce melanin under saprophytic conditions. Production of normal amounts of melanin by the isolate B on the yeast parasitic, stage, as detected by melanin ghost analysis and similar production of other virulence factors observed in this study support this latter hypothesis.

In fact, the experimental sporotrichosis animal model used in this study indicated a normal expression level of the virulence factors by isolate B. Surprisingly, we observed that it was more aggressive in mice than isolate A alone or the two isolates together. For instance, isolate B induced severe levels of inflammatory infiltrates and *S. brasiliensis* yeasts in the lungs and liver, in addition to histological alterations in all the organs examined. These infected animals also showed increased severity of hepatitis from day 35 to 49 after the inoculation, resulting in 10% mortality rate.

The spontaneous pericardial mineralization observed in the mice inoculated with isolate A or B is known as dystrophic cardiac calcinosis (DCC). This disease results from a cardiac injury that causes myocyte necrosis and is characterized by fibrosis and the formation of calcified plaques in the pericardium.^28^ ^,^ ^29^ Since we used male mice and BALB/c strain, both of which are factors associated with susceptibility to DCC,^28^ ^,^ ^29^ the occurrence of this disease in our study might have been influenced by our gender and strain choice. In this study, the pericardial mineralization and fibrosis were probably associated with the *S. brasiliensis* infection. This hypothesis is based on the fact that the control mice did not presented this histological alteration and DCC was more severe in mice inoculated with the isolate B, which was the only that showed endocarditis and pericarditis and severe pneumonia rich in macrophages associated to the *S. brasiliensis* infection. Lung macrophages^30^ and pericarditis caused by viral and bacterial infections^30^ are involved in the development of DCC in mice, reinforcing our hypothesis. The absence of pericardial mineralization lesion only in the mice infected with the combination of isolates A and B may be due to the absence of inflammatory lesions in the heart caused by the association of these two *S. brasiliensis* isolates.

The absence of *S. brasiliensis* yeasts in the examined organs of the mice inoculated with isolate A alone or A and B together, the low fungal load in the infected organs of the mice inoculated with isolate B, and the presence of well-organized granulomas in the tissues of all the groups infected with these isolates indicate an efficient immune response by BALB/c mice to the infection by all the *S. brasiliensis* strains tested. These results are in accordance with the feline model applied by Miranda and colleagues,^26^ which revealed that experimental sporotrichosis resulted in well-organized granulomas, low fungal loads, localized cutaneous lesions and good general condition of the cats.
